# Mapping the evidence on artificial intelligence–based clinical decision support systems for undergraduate nursing students: a scoping review protocol

**DOI:** 10.11124/JBIES-26-00065

**Published:** 2026-07-28

**Authors:** Francesco Scerbo, Gabriele Caggianelli, Simone Ciucciarelli, Giovanni Cangelosi, Valentina Vanzi, Rocco Mazzotta

**Affiliations:** 1 Department of Biomedicine and Prevention, University of Rome Tor Vergata, Rome, Italy; 2 Center of Excellence for Nursing Culture and Research, Order of Nursing Professions of Rome, Rome, Italy; 3 JBI Italy Evidence-Based Practice and Health Research Center, Rome, Italy; 4 Department of Healthcare Professions, Azienda Ospedaliera Complesso Ospedaliero San Giovanni Addolorata, Rome, Italy; 5 School of Pharmacy, Experimental Medicine and “Stefania Scuri” Public Health Department, University of Camerino, Camerino, Italy

**Keywords:** artificial intelligence, clinical decision support systems, clinical reasoning, nursing education, undergraduate nursing students

## Abstract

**Objective:**

This scoping review will map the scope and nature of the available evidence on the use of artificial intelligence–based clinical decision support systems (AI-CDSSs) for undergraduate nursing students.

**Introduction:**

AI is increasingly integrated into nursing education to support students’ clinical reasoning and decision-making. Yet the literature on AI-CDSSs remains fragmented and heterogeneous, with variability in technologies, educational applications, and reported outcomes, as well as concerns regarding bias and ethical implications. To date, no comprehensive synthesis has mapped the evidence on AI-CDSS use for undergraduate nursing students.

**Eligibility criteria:**

This review will map empirical research involving undergraduate nursing students using AI-based tools to support clinical reasoning, diagnostic reasoning, prioritization, or the nursing process. It will consider sources published in English, Spanish, French, Italian, or Chinese, provided that the title and abstract are in English and the record is considered relevant. Searches will be limited to sources published from 2017 onward, aligning with the rapid evolution and only recent widespread availability of modern generative AI tools.

**Methods:**

This review will follow the JBI methodology for scoping reviews. Searches will be conducted in PubMed, Scopus, CINAHL (EBSCOhost), the Cochrane Library, ERIC (EBSCOhost), and Web of Science Core Collection, while gray literature will be retrieved from ProQuest Dissertations and Theses Global (ProQuest), OpenAlex, Google Scholar, and professional body and regulatory sources. Screening will be performed independently by 2 reviewers, and data will be synthesized descriptively and thematically. Reporting will be documented according to the Preferred Reporting Items for Systematic Reviews and Meta-Analyses extension for Scoping Reviews (PRISMA-ScR).

**Review registration:**

OSF https://osf.io/eh9bt/

## Introduction

Artificial intelligence (AI) is increasingly shaping contemporary health care, supporting clinical decision-making through advanced data analysis, predictive modeling, and real-time recommendations.^1^ As AI-driven technologies become embedded in clinical infrastructures, these systems are beginning to influence professional workflows and redefine the competencies required of future health professionals.^1^ In parallel, nursing education is undergoing a profound transformation in response to the digitization of health care. Educators are increasingly exploring how AI-enhanced tools can facilitate the acquisition of clinical reasoning, diagnostic judgment, and decision-making competencies, which are foundational to safe and effective nursing practice.^2^-^5^

Major international professional and regulatory organizations—including the World Health Organization, International Council of Nurses, National League for Nursing, American Nurses Association, American Association of Colleges of Nursing, and European Federation of Nurses Associations—have emphasized the need to integrate AI literacy, informatics competencies, and digital professionalism into undergraduate nursing programs.^6^-^13^ These position statements collectively underscore that future nurses must be prepared not only to use AI-enabled systems but also to critically appraise their outputs, understand their limitations, and participate in their ethical and responsible implementation.

While these recommendations establish a clear institutional mandate for AI’s integration into nursing curricula, translating them into meaningful educational practice presents particular challenges for programs for undergraduate students. Unlike their postgraduate counterparts, who typically build upon existing clinical experience to refine advanced practice competencies, undergraduate nursing students are novice learners navigating the crucial initial transition from theoretical knowledge to practical clinical judgment. As such, they frequently encounter difficulties in synthesizing complex patient information, prioritizing care, and applying clinical knowledge in dynamic contexts,^14^-^16^ and they remain particularly susceptible to high cognitive loads during the decision-making process.^17^

Traditional educational strategies, such as case studies, simulation, and supervised clinical practice, remain essential but may not always offer the individualized, adaptive, and immediate feedback required to support the development of higher-order cognitive skills. Within this context, AI-based clinical decision support systems (AI-CDSSs) are emerging as educational tools with potential relevance for nursing education.^18^,^19^ These systems, which include predictive algorithms, adaptive simulations, and large language model–based applications, can support learners by guiding diagnostic pathways, highlighting potential errors, promoting reflective thinking, and providing structured, data-driven feedback. Furthermore, AI-CDSSs may contribute to standardizing learning experiences, reducing variability in clinical exposure, and supporting competency-based education models.

The gray literature highlights the growing institutional commitment to AI’s integration into nursing education. Recent frameworks developed by the American Association of Colleges of Nursing (AACN) and Governa.ai outline practical applications of AI-CDSSs for student learning, including their use in virtual clinical experiences, formative assessment, and personalized learning pathways.^20^,^21^ These documents emphasize that AI-enhanced tools can complement traditional teaching strategies by offering scalable, flexible, and interactive learning environments. Furthermore, these frameworks highlight the potential of AI to support the faculty in curriculum design, learner analytics, and the identification of students who may require additional support.

Despite these opportunities, concerns persist regarding algorithmic bias, which can actively propagate health care disparities. A major factor is sampling bias, which occurs when AI models are trained predominantly on datasets that fail to represent the broader population, often disproportionately reflecting specific racial groups, genders, or age demographics. Over-relying on such non-representative models compromises their reliability, ultimately leading to inequitable care and inaccurate clinical predictions for underrepresented populations, along with poor interpretability, cognitive dependence, and unintended consequences of AI in clinical contexts.^22^-^25^ These issues are particularly relevant in educational settings, where students may lack the experience needed to critically evaluate AI-generated outputs or to recognize when such outputs may be inaccurate or inappropriate. The risks of over-reliance on AI tools, reduced development of independent clinical judgment, and reinforcement of biased or incomplete information represent important challenges that must be addressed through thoughtful pedagogical design and appropriate supervision. In that context, ethical considerations, including transparency, accountability, data privacy, and the need for explainable AI, are increasingly recognized as essential components of AI-enhanced nursing education.

Although interest in AI-CDSSs within nursing education is growing, the existing literature remains fragmented and heterogeneous. Studies differ widely in terms of technologies described, educational purposes, learning contexts, and reported outcomes. A preliminary search of PubMed, the Cochrane Database of Systematic Reviews, *JBI Evidence Synthesis*, protocol registries (eg, PROSPERO, OSF, INPLASY), and preprint servers (eg, medRxiv), using keywords such as *artificial intelligence, clinical decision support*, and *nursing students*, was conducted in January 2026, and no current or in-progress scoping reviews or systematic reviews on the topic were identified. Existing reviews tend to focus broadly on digital health, virtual simulation, or AI in nursing practice, without isolating the unique pedagogical, cognitive, and ethical implications of AI-CDSSs for nursing students. Focusing exclusively on this group is methodologically essential, as assuming that clinical reasoning is identical across health care professions overlooks fundamental epistemic differences.^26^ Given the identified gap, a comprehensive synthesis is warranted.

This scoping review will follow the JBI methodology^27^ and will map the scope and nature of the available evidence on the use of AI-CDSSs for undergraduate nursing students. Specifically, the review will explore the types of AI-CDSSs used, their educational applications, the outcomes reported, and the challenges and ethical considerations associated with their integration into teaching and learning processes. By examining the existing literature and identifying knowledge gaps, this review will inform educators, researchers, and policymakers, and support the evidence-informed and responsible integration of AI-CDSSs into nursing education.

## Review questions

What is the extent, range, and nature of the literature regarding the use of AI-CDSSs for undergraduate nursing students within nursing education settings?

The primary research question will be supported by the following subquestions:

- Which types of AI-CDSSs and related AI-based tools are described in the literature for use with undergraduate nursing students?
- For what educational purposes and learning activities are AI-CDSSs used with undergraduate nursing students (eg, clinical reasoning, diagnostic reasoning, decision-making, care prioritization, nursing process)?
- In which educational settings are AI-CDSSs used with undergraduate nursing students (eg, classroom-based learning, simulation, clinical placements, virtual learning environments)?
- What types of outcomes (stratified into cognitive, affective, performance-based, implementation-related domains) are descriptively reported in relation to the use of AI-CDSSs for undergraduate nursing students, and how are they measured?
- What challenges and considerations are reported when integrating AI-CDSSs into programs for undergraduate nursing students?

## Eligibility criteria

### Participants

This review will consider studies that include prelicensure, preregistration, and undergraduate nursing students enrolled in nursing education programs (depending on country-specific educational structures and including equivalent entry-to-practice programs). Registered nurses, postgraduate nursing students, and other health professionals will be excluded. Studies including mixed populations will be excluded unless data specific to undergraduate nursing students are clearly identifiable and reported separately.

### Concept

This review will consider studies that explore AI, defined as “the ability of algorithms encoded in technology to learn from data so that they can perform automated tasks without every step in the process having to be programmed explicitly by a human.”^13^^(p.xi)^ For the purposes of inclusion, AI systems must involve dynamic processing of data to generate recommendations, predictive insights, or adaptive learning paths, differentiating them from static algorithms or standard rule-based software. These definitions will be operationalized by considering only studies where these tools actively interact with students to support clinical reasoning, diagnostic reasoning, decision-making, prioritization, or the nursing process.

This review will consider studies involving AI-CDSSs and related AI tools (eg, predictive algorithms, large language models, chatbots, adaptive simulations, intelligent tutoring systems) that involve direct interaction with nursing students. Direct interaction is defined as iterative engagement between the student and the AI tool (eg, back-and-forth prompt revision, adaptive dialog, dynamic feedback loops), rather than a single static search query. This interaction may occur directly through stand-alone applications or indirectly through AI-embedded simulation platforms and instructor-mediated interfaces, provided the student is the end user of the AI-generated feedback. Backend analytic systems used solely by the faculty will be excluded. Furthermore, passive digital interventions (eg, static e-learning modules) and conventional non-AI clinical databases will be excluded. To align with the CDSS framework, tools such as large language models or chatbots will be considered only if they have a clear clinical decision support function (eg, guiding diagnostic reasoning, care planning, patient prioritization), excluding generic question-answering tools or broad teaching aids.

Sources will also be considered only if they report outcomes associated with the use of AI-CDSSs, stratified into the following domains: cognitive (eg, knowledge acquisition, clinical reasoning, critical thinking), affective (eg, attitudes toward AI, confidence, satisfaction), performance-based (eg, diagnostic accuracy, clinical competency, task completion), and implementation-related (eg, usability, adoption rates, integration into educational workflows). Additionally, this review will consider sources reporting challenges and considerations associated with AI-CDSS integration (eg, algorithmic bias, data privacy, cognitive dependence, interpretability, ethical implications).

### Context

The review will consider studies that address nursing education settings, including academic, clinical, and simulated learning environments (eg, classrooms, simulation laboratories, clinical placements), across all geographical locations.

### Types of sources

This review will consider empirical studies (quantitative, qualitative, mixed methods), reviews, theses, conference proceedings, policy documents, clinical guidance, position statements, and nursing education guidelines from professional organizations. In addition, gray literature will be considered for inclusion in the proposed scoping review. Non-empirical publications such as editorials, commentaries, and individual opinion papers (with the exception of official professional guidelines and position statements), as well as studies focused solely on technical performance or patient education, will be excluded. However, non-empirical sources (eg, policy documents, professional guidelines, reviews) will be considered as supplementary material to map the contextual, ethical, and regulatory landscape of AI-CDSS integration.

## Methods

The proposed scoping review will be conducted in accordance with the JBI methodology for scoping reviews^27^ and reported in line with the Preferred Reporting Items for Systematic Reviews and Meta-Analyses extension for Scoping Reviews (PRISMA-ScR).^28^ The protocol has been registered in OSF (https://osf.io/eh9bt/).

### Search strategy

An initial limited search of PubMed and CINAHL was undertaken to identify sources of evidence on the topic. The text words contained in the titles and abstracts of relevant articles, and the index terms used to describe the articles, were used to develop a full search strategy for PubMed (see Appendix I). The search strategy, including all identified keywords and index terms, will be adapted for each included information source. Additional keywords will include terms related to clinical judgment and intelligent tutoring systems, given their relevance in contemporary nursing education and AI-supported decision-making (eg, clinical judgment, clinical decision-making, clinical competence, intelligent tutoring system*, AI tutor*). The reference lists of articles included in the review will be screened for additional papers.

Sources published in English, Spanish, French, Italian, and Chinese will be considered for inclusion. These languages were selected based on the language proficiencies available within the review team, ensuring that all translated content will be rigorously verified rather than relying solely on unverified automated translation. For non-English records (Spanish, French, Italian, Chinese), initial title and abstract screening will be conducted by bilingual members of the research team or by utilizing automated translation tools, with each translated output verified by a bilingual team member for accuracy before deciding its eligibility. If a non-English full text is selected for inclusion in the review, data extraction will be performed collaboratively by native-speaking researchers or with the assistance of professional academic translation services to ensure accuracy and rigor. Searches will be limited to sources published from 2017 onward, corresponding to the advent of modern generative AI architectures and their general availability.

The databases to be searched include PubMed, Scopus, CINAHL (EBSCOhost), the Cochrane Library, ERIC (EBSCOhost), and Web of Science Core Collection. Sources of gray literature to be searched include ProQuest Dissertations and Theses Global (ProQuest), OpenAlex, Google Scholar (first 200 results), and professional body and regulatory sources (World Health Organization, International Council of Nurses, National League for Nursing, American Nurses Association, AACN, European Federation of Nurses Associations, Healthcare Information and Management Systems Society, Health Education England).

### Study selection

Following the search, all identified records will be collated and imported into EndNote v.2025.3.1 (Clarivate Analytics, PA, USA) and duplicates removed before they are uploaded to Rayyan (Rayyan Systems Inc., Cambridge, MA, USA). Titles and abstracts will be screened by 2 reviewers independently for assessment against the eligibility criteria for the review. A pilot test of 10% of the records will be undertaken to ensure consistency in the application of the criteria, with formal screening commencing upon achieving >75% agreement between reviewers. Potentially relevant records will be retrieved in full and their citation details imported into the JBI System for the Unified Management, Assessment and Review of Information (JBI SUMARI; JBI, Adelaide, Australia).^29^ Then, the full text of citations will be assessed in detail against the eligibility criteria by 2 reviewers independently. Reasons for exclusion of full-text papers that do not meet the eligibility criteria will be recorded and reported in the scoping review. Any disagreements that arise between the reviewers will be resolved through discussion or with a third reviewer. The results of the search will be reported in full in the final scoping review and presented in a PRISMA flow diagram.^30^

### Data extraction

Data will be extracted from sources included in the scoping review by 2 reviewers independently using a data extraction tool developed by the reviewers based on the *JBI Manual for Evidence Synthesis*.^27^ The data extracted will include specific details about AI transparency and explainability (eg, interpretability features, availability of model rationale, user-facing explanations), given their centrality in AI ethics and educational safety, as well as crucial educational safety variables, including the risk of hallucination or factual inaccuracy, the required supervision model, whether AI outputs were verified by the faculty or clinical experts, and related trust or bias concerns. Beyond that, additional extracted data will include bibliographic details, study characteristics, participant demographics, AI-CDSS features, educational aims, outcome measures, challenges, and ethical considerations. A draft extraction tool is provided (see Appendix II). The draft extraction tool will be modified and revised as necessary during the process of extracting data from each included source. Modifications will be detailed in the full scoping review. Any disagreements that arise between the reviewers will be resolved through discussion or with a third reviewer. In cases where data are missing or incomplete, authors of papers will be contacted up to 2 times to obtain the necessary information. If no response is received, the potential impact on the findings will be considered during data synthesis.

### Data analysis and presentation

The proposed scoping review will categorize AI types based on their underlying technological architecture (eg, machine learning, large language models) and their level of autonomy. Data will be synthesized descriptively (frequencies, distributions) and thematically (conceptual categories related to educational use; outcomes stratified into cognitive, affective, performance-based, implementation-related domains; challenges). Data on concerns regarding bias and ethical implications will be explicitly sought during extraction and incorporated into the thematic analysis to highlight reported risks and mitigation strategies. Results will be presented in tables, figures, and narrative summaries. A conceptual map will be developed to visually synthesize relationships between AI technology types, educational functions, outcomes, and reported challenges. This mapping will support a clearer understanding of how AI-CDSSs are being integrated into undergraduate nursing education.

## Author contributions

FS, RM, SC: protocol conception, design, search strategy, manuscript drafting. FS, RM, SC: review concept development, methodological design. FS, VV: manuscript drafting, critical revision of protocol. G Caggianelli, G Cangelosi: methodological and search strategy oversight, critical review of final manuscript. All authors approved the final version to be published and agree to be accountable for all aspects of the work to ensure its accuracy and integrity.

## Appendix I: Search strategy

**Table:**

PubMed		
Search conducted: April 3, 2026		
Search	Query	Records retrieved
#1	“student nurse*”[tiab] OR “nursing student*”[tiab] OR “students, nursing”[MeSH Terms] OR “prelicensure nursing”[tiab] OR “pre-licensure nursing”[tiab] OR “preregistration nursing”[tiab] OR “undergraduate nurs*”[tiab]	46,734
#2	“clinical decision support” [tiab] OR ((“decision”[tiab] OR “decision s”[tiab] OR “decisions”[tiab] OR “decisive”[All Fields] OR “decisively”[tiab]) AND (“support”[tiab] OR “support s”[tiab] OR “supported”[tiab] OR “supporter”[tiab] OR “supporter s”[tiab] OR “supporters”[tiab] OR “supporting”[tiab] OR “supportive”[tiab] OR “supportiveness”[tiab] OR “supports”[tiab]) AND “system*”[tiab]) OR “cdss”[tiab] OR “decision support systems, clinical”[MeSH Terms] OR “decision support”[tiab] OR “patient decision support”[tiab] OR “computer assisted decision”[tiab] OR “decision making processes”[tiab] OR (“decision making”[MeSH Terms] OR (“decision”[tiab] AND “making”[tiab]) OR “decision making”[tiab]) OR “decision making, computer assisted”[MeSH Terms] OR “clinical decision making”[MeSH Terms]	737,560
#3	“artificial intelligence”[tiab] OR “artificial intelligence”[MeSH Terms] OR “machine learning”[tiab] OR “deep learning”[tiab] OR “predictive algorithm”[tiab] OR “large language model”[tiab] OR “llm”[tiab] OR “large language models”[MeSH Terms] OR “generative artificial intelligence”[MeSH Terms] OR “generative ai”[tiab] OR “chatbot”[tiab]	503,795
#4	#2 AND #3	62,547
#5	#1 AND #4	55

## Appendix II: Draft data extraction instrument

**Table:**

Section	Data item	Description/example of data to extract
**Source information**	Authors	List all named
	Year of publication	
	Title of study	
	Country of origin	Or region
	Source type	Journal article, thesis, conference proceeding, policy document, gray literature
	Type of evidence source	Empirical study, review, educational report, gray literature
**Study characteristics**	Study design	Qualitative, quantitative, mixed methods, descriptive, quasi-experimental, etc
	Methods of data collection	Survey, interview, observation, focus group, document review, etc
	Data analysis methods	Descriptive statistics, inferential analysis, thematic analysis, content analysis
**Population**	Type of participant	Pre-licensure, preregistration, or undergraduate nursing students; specify year/level if available
	Number of participants	
	Eligibility criteria	
**Context**	Educational setting	Academic, clinical, simulation lab, blended environment
	Organizational setting	University, hospital, simulation center, digital platform
**Concept: AI/AI-CDSS intervention**	Type of AI technology	ML/DL models, predictive analytics, LLMs, chatbots, adaptive simulations, expert systems
	Function of tool	Clinical reasoning support, diagnostic reasoning, prioritization, nursing process support, structured feedback
	Technical characteristics	Level of autonomy (assistive/semi-autonomous/autonomous), interaction mode (text, voice, simulation), integration with INKA/ICNP/NIC/NOC
**Educational aims**	Target competencies	Diagnostic accuracy, critical thinking, problem-solving, self-efficacy, clinical judgment, cognitive load, anxiety, satisfaction
	Alignment with frameworks	AACN, WHO, ICN, EFN, etc
**Findings**	Quantitative results	Outcomes measured (stratified into cognitive, affective, performance-based, implementation domains), instruments used, main results
	Qualitative results	Themes, perceptions of students and educators
	Overall educational impact	Improvements, no change, mixed results
**Challenges and ethics**	Reported issues	Algorithmic bias, interpretability, cognitive dependence, cognitive load, supervision needs, privacy/security concerns, organizational barriers, hallucination/factual inaccuracy risk, faculty verification of outputs, transparency, trust concerns
**Additional information**	Tool development/validation	Domains assessed, validation type, reliability statistics
	Reviewer notes	Comments, clarifications, observations

AACN, American Association of Colleges of Nursing; AI, artificial intelligence; AI-CDSS, artificial intelligence–based clinical decision support system; DL, deep learning; EFN, European Federation of Nurses Associations; ICN, International Council of Nurses; ICNP, International Classification for Nursing Practice; INKA, International Nursing Knowledge Association; LLM, large language model; ML, machine learning; NIC, Nursing Interventions Classification; NOC, Nursing Outcomes Classification; WHO, World Health Organization.
